# *De novo *sequencing and analysis of the American ginseng root transcriptome using a GS FLX Titanium platform to discover putative genes involved in ginsenoside biosynthesis

**DOI:** 10.1186/1471-2164-11-262

**Published:** 2010-04-24

**Authors:** Chao Sun, Ying Li, Qiong Wu, Hongmei Luo, Yongzhen Sun, Jingyuan Song, Edmund MK Lui, Shilin Chen

**Affiliations:** 1 Institute of Medicinal Plant Development (IMPLAD), Chinese Academy of Medical Sciences, No.151, Malianwa North Road, Haidian District, Beijing 100193, China; 2 Department of Physiology and Pharmacology, The University of Western Ontario, Dental Sciences Building, London, ON N6A 5C1, Canada

## Abstract

**Background:**

American ginseng (*Panax quinquefolius *L.) is one of the most widely used herbal remedies in the world. Its major bioactive constituents are the triterpene saponins known as ginsenosides. However, little is known about ginsenoside biosynthesis in American ginseng, especially the late steps of the pathway.

**Results:**

In this study, a one-quarter 454 sequencing run produced 209,747 high-quality reads with an average sequence length of 427 bases. *De novo *assembly generated 31,088 unique sequences containing 16,592 contigs and 14,496 singletons. About 93.1% of the high-quality reads were assembled into contigs with an average 8-fold coverage. A total of 21,684 (69.8%) unique sequences were annotated by a BLAST similarity search against four public sequence databases, and 4,097 of the unique sequences were assigned to specific metabolic pathways by the Kyoto Encyclopedia of Genes and Genomes. Based on the bioinformatic analysis described above, we found all of the known enzymes involved in ginsenoside backbone synthesis, starting from acetyl-CoA via the isoprenoid pathway. Additionally, a total of 150 cytochrome P450 (CYP450) and 235 glycosyltransferase unique sequences were found in the 454 cDNA library, some of which encode enzymes responsible for the conversion of the ginsenoside backbone into the various ginsenosides. Finally, one CYP450 and four UDP-glycosyltransferases were selected as the candidates most likely to be involved in ginsenoside biosynthesis through a methyl jasmonate (MeJA) inducibility experiment and tissue-specific expression pattern analysis based on a real-time PCR assay.

**Conclusions:**

We demonstrated, with the assistance of the MeJA inducibility experiment and tissue-specific expression pattern analysis, that transcriptome analysis based on 454 pyrosequencing is a powerful tool for determining the genes encoding enzymes responsible for the biosynthesis of secondary metabolites in non-model plants. Additionally, the expressed sequence tags (ESTs) and unique sequences from this study provide an important resource for the scientific community that is interested in the molecular genetics and functional genomics of American ginseng.

## Background

**Figure 1:**
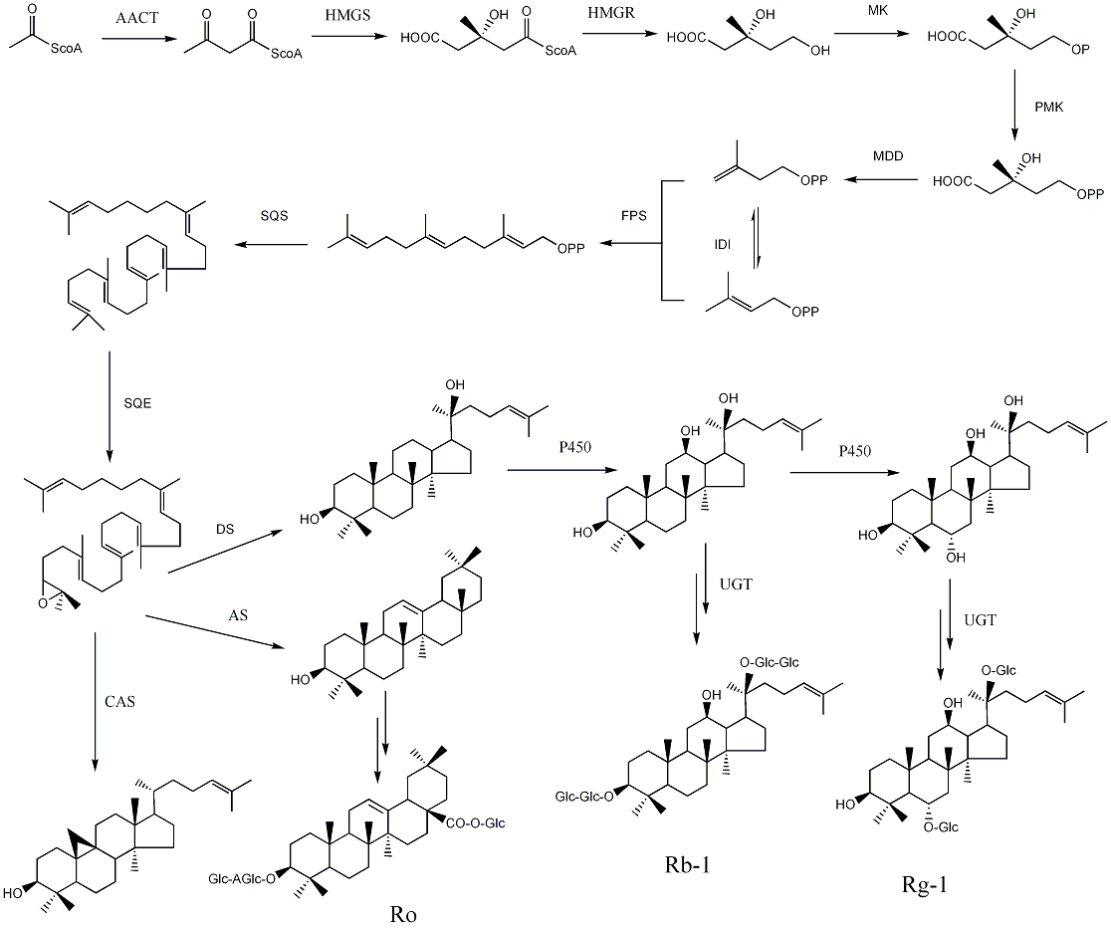
**Putative ginsenoside biosynthetic pathway in American ginseng**. AACT, acetyl-CoA acetyltransferase; AS, β-amyrin synthase; CAS, cycloartenol synthase; DS, dammarenediol-II synthase; FPS, farnesyl diphosphate synthase; GT, glycosyltransferase; HMGR, HMG-CoA reductase; HMGS, HMG-CoA synthase; IDI, isopentenyl diphosphate isomerase; MDD, mevalonate diphosphate decarboxylase; MK, mevalonate kinase; P450, cytochrome P450; PMK, phosphomevalonate kinase; Rb-1, ginsenoside Rb-1; Rg-1, ginsenoside Rg-1; Ro, ginsenoside Ro; SQE, squalene epoxidase; and SQS, squalene synthase.

American ginseng (*Panax quinquefolius *L.) is a perennial understory herb from the Araliaceae family, which is native to the eastern forests of North America [1]. It is one of the most extensively used medicinal plants in both East Asia and the West as a remedy or adaptogen to promote vitality, enhance physical performance, and increase resistance to stress and aging [2,3]. The major bioactive components of American ginseng are the triterpene saponins known as ginsenosides. To date, more than 30 ginsenosides have been isolated from American ginseng and are classified into two main groups, the dammarane type and the oleanane type, based on the structures of their aglycones. The major ginsenosides are the dammarane type, and include Rb1, Rc, Rd, Re, and Rg1. The oleanane type is represented by only one saponin, Ro, which is a minor component in American ginseng [4,5]. Each ginsenoside reportedly has different pharmacological effects, including nervous system regulation, immune system modulation, and anticancer, antioxidant, and antihypertensive activities [6-9]. Ginsenosides are synthesized by the isoprenoid pathway and share the same precursor, 2,3-oxidosqualene, with sterol [10]. Due to the biological importance of sterol, the common steps in its conversion from acetyl-CoA to 2,3-oxidosqualene have been widely studied in many plant species. The cyclization of oxidosqualene is the branch point for the biosynthesis of ginsenosides and phytosterols. The 2,3-oxidosqualene cyclases (OSCs) that synthesize β-amyrin [11] and dammarenediol-II [12,13] have been characterized in *Panax ginseng*; however, the portion of the pathway that lies downstream of cyclization remains largely unknown. According to the proposed pathway (Shown in Figure 1), some specific CYP450s and UDP-glycosyltransferases (UGTs) may catalyze the conversion of dammarenediol-II or β-amyrin to various ginsenosides [14]. To date, no CYP450s or UGTs involved in ginsenoside biosynthesis have been identified from American ginseng or other ginsenoside-producing plants.

The sequencing and analysis of ESTs are the primary tools for the discovery of novel genes, especially in non-model plants for which full genome sequencing is not economically feasible. EST sequencing represents a rapid and relatively economical method for analyzing the transcribed region of the genome [15]. Furthermore, EST analyses have identified the genes involved in plant secondary metabolism. For example, a licorice-amyrin 11-oxidase gene was successfully identified by the analysis of a collection of ESTs from the stolons of *Glycyrrhiza uralensis*; this gene encodes a CYP450 that plays a key role in the biosynthesis of the triterpene sweetener glycyrrhizin [16]. Additionally, a dammarenediol synthase was functionally characterized from the ESTs generated from a *P. ginseng *flower cDNA library [13]. ESTs can also be used for other functional genomic projects, including gene expression profiling, microarrays, molecular markers, and physical mapping [15,17].

Over the last few years, next-generation sequencing (NGS) technologies have led to a revolution in genomics and genetics and provided cheaper and faster delivery of sequencing information [18,19]. The first commercial NGS platform, 454 GS20 http://www.454.com, was released in 2005 and produces about 200,000 reads with an average read length of 100 bases per run [20,21]. Since then, 454 sequencing technology has experienced a rapid improvement in throughput, read length, and accuracy. Now, the newest 454 sequencing platform, the GS FLX Titanium, can generate one million reads with an average length of 400 bases at 99.5% accuracy per run. To date, the 454 pyrosequencing technique is the most widely used NGS technology for the *de novo *sequencing and analysis of transcriptomes in non-model organisms.

Despite the commercial and medicinal importance of American ginseng, little genomic research has been performed with this species. In this study, with the Roche GS FLX Titanium platform, we obtained more than 200,000 high-quality (HQ) reads from a cDNA library generated from an American ginseng root. Those reads were assembled into 31,088 unique transcripts, containing 16,592 contigs and 14,496 singletons. The average lengths of the HQ reads and the contigs were comparable to those generated from an American ginseng root cDNA library in our previous study using the Sanger method [22]. Bioinformatic analysis indicated that all genes encoding enzymes involved in the biosynthesis of the ginsenoside backbone existed in the transcriptome of the American ginseng root. Furthermore, a few candidate genes putatively responsible for ginsenoside backbone modifications were screened out of a gene pool containing 150 CYP450 and 235 UGT unique sequences. To the best of our knowledge, this study is the first exploration to discover the genes responsible for triterpene saponin biosynthesis through the analysis of large-scale ESTs produced from a next-generation sequencer. Additionally, the method described here can be widely applied to the profiling of transcriptomes, facilitating the discovery of novel genes in other non-model organisms.

## Results

### Sequencing and *de novo *assembly of 454 ESTs

**Figure 2:**
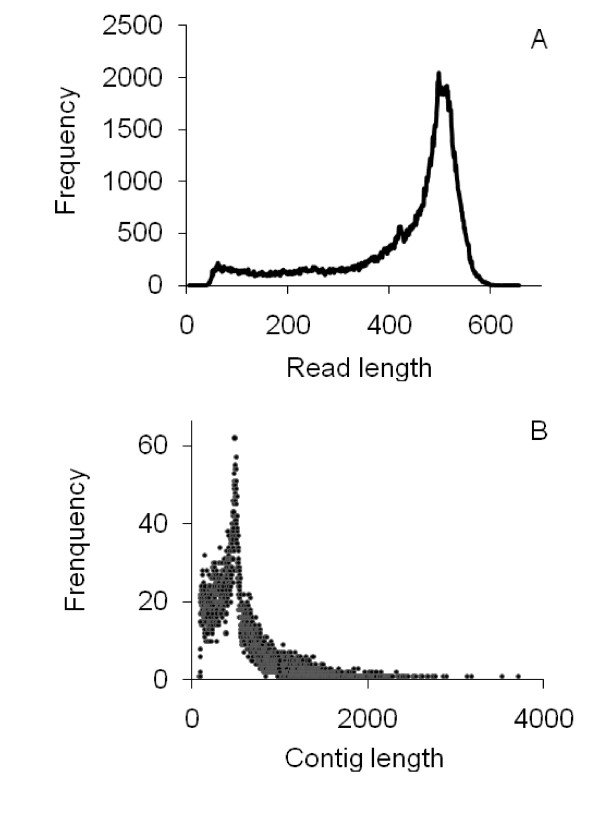
**Size distribution of the 454 HQ reads and the contigs assembled from them**. A) 454 HQ reads; B) contigs.

A cDNA library constructed by SMART technology from the total RNA of an American ginseng root was subjected to a one-quarter plate run with the 454 GS FLX Titanium platform. This one-quarter run produced 209,747 HQ reads with an average sequence length of 427 bases (SD = 128, range = 20-1135). Of the HQ reads, 90.5% contained more than 200 bases, whereas 73.5% had more than 400 bases. The size distribution of the reads is shown in Figure 2-A. After trimming the adapter sequences and removing the short sequences of less than 50 bases, 207,311 reads remained for assembly with an average length of 422 ± 123 bases. A total of 89.7 Mb of HQ sequence data were generated, of which 87.4 Mb (97.5% of the HQ sequence data) were used for assembly. All HQ reads were also deposited in the National Center for Biotechnology Information (NCBI) and can be accessed in the Short Read Archive (SRA) under the accession number SRX012184. An overview of the sequencing and assembly is given in Table 1.

**Table 1:** Overview of the sequencing and assembly

	Sequence (n)	Bases (bp)
**Sequencing**		
High-quality (HQ) reads	209,747	89,664,715
Average HQ read length	427 ± 128 bp	
Reads used in assembly	207,311	87,443,477
Average read length after trimming	422 ± 123 bp	

**Contigs**		
Reads assembled as contigs	195,251	
Number of contigs	16,592	8,722,777
Average length of contigs	526 ± 333 bp	
Range of contig length	92 - 3713 bp	
Depth on contigs	8.46	

**Singletons**		
Number of singletons	14,496	5,276,139
Average length of singletons	364 ± 166 bp	
Range of singleton length	50 -- 643 bp	

**Unique sequence**	31,088	13,998,916

The reads produced by the GS FLX Titanium platform were long enough for *de novo *assembly. Therefore, size-selected reads were assembled into 16,592 contigs by Roche Newbler software, while 14,496 reads remained as singletons. The sequencing coverage ranged from 1 to 3,102 fold, with an average 8-fold coverage. In total, 195,251 reads were assembled into contigs, accounting for 93.1% of the assembled reads. Contigs ranged from 92 to 3,713 bases, with an average size of 526 ± 333 bases. About 81.6% of the contigs were assembled from three or more reads. The size distribution for these contigs is shown in Figure 2-B.

### Functional annotation by sequence comparison with public databases

All unique sequences were first compared with the sequences in the non-redundant database (Nt) of the NCBI using the BLASTN algorithm and were then compared with the sequences in the three major public protein databases (listed in the Methods section) using the BLASTX algorithm. When the E-value cutoff was set at 10^-5^, a total of 21,684 unique sequences were annotated, which accounted for 69.8% of the total unique sequences. Under a more stringent condition (cutoff = 10^-10^), 19,779 unique sequences were annotated, which accounted for 63.6% of the total unique sequences (shown in Additional File 1).

Of all the contigs, a total of 16 contained more than 1,000 reads, which represented the most abundant transcripts in the 454 EST cDNA library (Additional File 2). The 16 most abundant transcripts included some that encoded abundant root proteins that were previously characterized in Chinese ginseng (*P. ginseng*) or American ginseng (*P. quinquefolius*), such as Ribonuclease-like storage protein, *P. quinquefolius*-specific abundant protein-like protein 1, and *P. ginseng *major latex-like protein (mlp151) [23,24]. The most abundant transcript, which had 3,102 reads, was annotated as "regulator of Ribonuclease-like protein 1," a possible inhibitor of the endonuclease activity of RNase. Interestingly, we also found an abundant transcript encoding RNase 1. It is not surprising that some transcripts encoding the enzymes involved in sugar and energy metabolism were highly expressed, because starch is the most abundant component of the American ginseng root, and these enzymes included sucrose synthase and 1, 4-alpha-glucan-branching enzyme. Some transcripts encoding peroxidases were also highly expressed, and these enzymes may play a role in resistance to abiotic or biotic stresses [25].

### Functional classification by KEGG

Functional classification and pathway assignment was performed by the Kyoto Encyclopedia of Genes and Genomes (KEGG). First, the 31,088 unique sequences were compared using BLASTX with an E-value cutoff of <10^-5 ^against the KEGG database. Of these unique sequences, 16,480 (53.0%) had significant matches in the database sequences. Among those, 4,097 unique sequences having enzyme commission (EC) numbers were assigned to metabolic pathways. As shown in Additional File 3, the KEGG metabolic pathways that were well represented by the American ginseng unique sequences were carbohydrate metabolism, amino acid metabolism, energy metabolism, and lipid metabolism. In the subclass of secondary metabolism, the greatest number of unique sequences was mapped to phenylpropanoid biosynthesis and limonene and pinene degradation. Ginsenosides belong to the terpenoid saponins, which share a common pathway from acetyl-CoA to 2,3-oxidosqualene with sterol; therefore, we focused more of our attention on sterol and terpenoid biosynthesis. Surprisingly, in the KEGG map of sterol biosynthesis (Shown in Additional File 4), most enzymes were mapped to transcripts in the 454 cDNA library and included all of the enzymes involved in sterol backbone synthesis and in brassinosteroid and stigmasterol biosynthesis. This result demonstrated the powerful ability of high-throughput sequencing to identify genes in metabolic pathways.

### Candidate enzymes involved in ginsenoside biosynthesis

#### OSCs and other known enzymes responsible for the biosynthesis of ginsenoside backbones

As shown in Figure 1, it is generally thought that ginsenosides are synthesized via the mevalonate pathway. Based on the KEGG pathway assignment, we found all of the genes encoding enzymes involved in ginsenoside backbone biosynthesis (Table 2). In most cases, more than one unique sequence was annotated as the same enzyme. Such unique sequences may represent different fragments of a single transcript, different members of a gene family, or both.

**Table 2:** Known genes involved in ginsenoside backbone biosynthesis

Gene Name	EC number	Unique sequence	EST number
*AACT*	2.3.1.9	contig02881, contig06048	204
*HMGS*	2.3.3.10	contig08477	23
*HMGR*	1.1.1.34	contig02603, contig02626, contig03619, contig06897, contig06899	62
*MK*	2.7.1.36	contig05022	21
*PMK*	2.7.4.2	contig04341, contig12148, FW1NBNE03GNFKM	8
*MDD*	4.1.1.33	contig06712	61
*IDI*	5.3.3.2	contig05719, contig10448	23
*FPS*	2.5.1.10	contig12444, contig15186	47
*SQS*	2.5.1.21	contig08056, contig08936, contig15530	32
*SQE*	1.14.99.7	contig00302, contig00303, contig02846, contig11541, contig15180, contig15497	173
*DS*		contig02334, contig03192, contig10031	36
*AS*		FW1NBNE03FS0YO(AS1), FW1NBNE03GOEZN(AS1), FW1NBNE03GJOPM(AS2)	3

**Figure 3:**
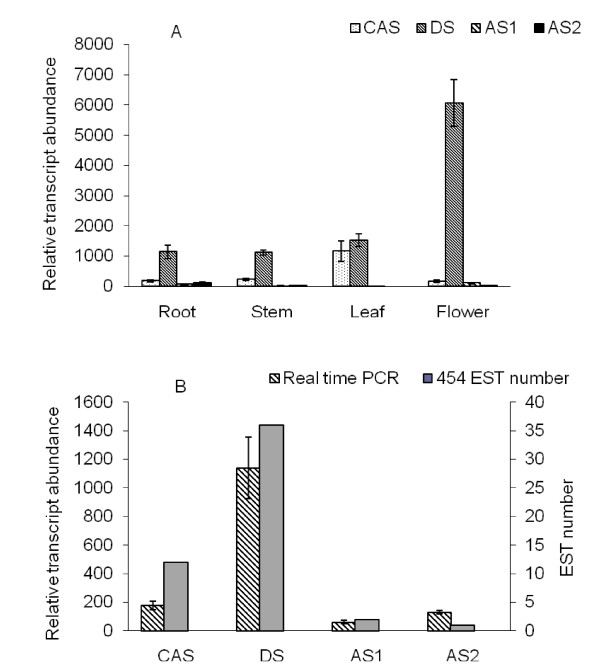
**Expression patterns of *OSC*s in different plant tissues**. A) *OSC *expression in the root, stem, leaf, and flower of American ginseng. B) Comparison of the relative abundance of *OSC *transcripts in American ginseng roots resulting from real-time PCR and EST counting, respectively.

The cyclization of 2,3-oxidosqualene is the branch point of sterol and ginsenoside biosynthesis and controls the carbon flux through the branched biosynthetic pathways. Four OSC genes of American ginseng, *cycloartenol synthase *(*CAS*) [26], *dammarenediol-II synthase *(*DS*) [12,13], *β-amyrin synthase 1 *(*AS1*), and *β-amyrin synthase 2 *(*AS2*) [11], exist in the 454 cDNA library. We investigated their expression levels in different plant tissues by real-time PCR. As shown in Figure 3-A, *DS *was highly expressed in all selected tissues, and its highest expression was found in flowers. In contrast, *CAS *expression was the lowest in flowers. Furthermore, *CAS *was expressed at a lower level than *DS *in other tissues. Compared with *DS *and *CAS*, *AS1 *and *AS2 *displayed a low level of expression in all tested tissues. In fact, AS2 transcripts were not detected in the leaves. We also compared the results from the real-time PCR and EST number counting (shown in Figure 3-B). Their significant agreement indicated that the abundance of the 454 sequences from the non-normalized cDNA library closely mirrors the actual expression level, although amplification can introduce some biases.

#### Cytochrome P450s

**Figure 4:**
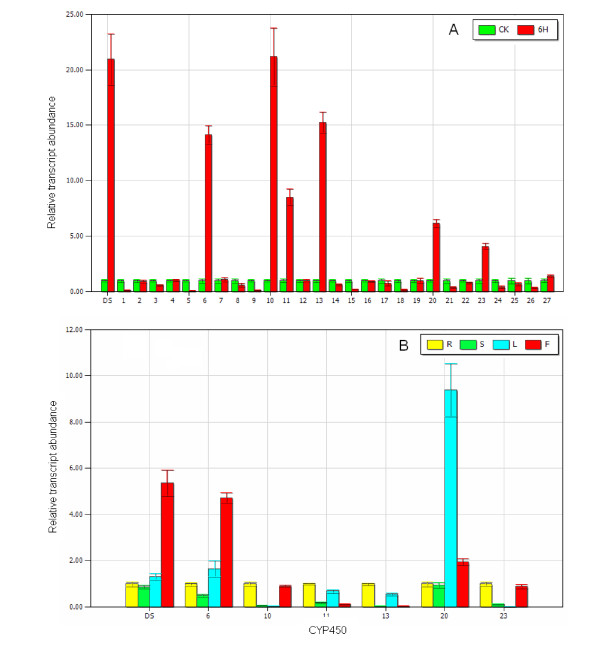
**Real time PCR analysis of *CYP450*s in MeJA-treated materials and different plant tissues**. CK represents the uninduced material; 6H represents the material treated for 6 h with MeJA; and DS represents dammarenediol-II synthase. The corresponding contigs represented by numbers 1 - 27 are listed in Additional File 6. A) Changes in the gene expression of *CYP450*s induced by MeJA. B) The gene expression of *CYP450*s in different tissues.

Cytochrome P450 proteins are the largest family of plant proteins and catalyze most of the oxidation steps in plant secondary metabolism [27,28]. In the biosynthetic pathway of dammarane-type ginsenosides, two steps are catalyzed by CYP450s: the conversion from dammarane to protopanaxadiol and the conversion from protopanaxadiol to protopanaxatriol. A total of 150 unique sequences, 92 contigs and 58 singletons, were annotated as CYP450s (Additional File 5). To find the CYP450s involved in dammarane-type ginsenoside biosynthesis, these unique sequences were further screened according to classification, abundance, MeJA inducibility, and tissue-specific expression. As shown in Figure 1, dammarane and protopanaxadiol, which are triterpenes, have a structure that is similar to plant sterols. Thus far, all known triterpenes and sterol hydroxylases have been classified into two clans: the CYP71 clan and the CYP85 clan [16,29-32]. *DS *had 36 ESTs, and we estimated that the *CYP450*s of moderate abundance were more likely to be involved in ginsenoside biosynthesis. Therefore, a total of 27 *CYP450*s containing 4-100 reads, which belonged to the CYP71 and CYP85 clans, were chosen for the MeJA inducibility experiment. The plant signaling compound MeJA induces or increases the biosynthesis of many secondary metabolites [33]. It has been reported that MeJA stimulates ginsenoside production in cultured ginseng cells [34] and adventitious roots [35], and up-regulates the genes involved in the biosynthesis of dammarane-type ginsenosides, such as *SS *(*squalene synthase*), *Squalene epoxidase *(*SE*), *DS *[13,34]. As shown in Figure 4-A, *DS *expression increased about 6-fold after MeJA treatment. Six *CYP450*s were up-regulated by MeJA, while eight *CYP450*s were down-regulated. The expression pattern of the six *CYP450*s in different tissues was then determined by real-time PCR. As shown in Figure 4-B, the highest level of *DS *expression was in the flower, while its expression in other three tissue types was much lower. Of all six *CYP450*s, only contig00248 had a similar tissue-specific expression pattern as *DS*, indicating that contig00248 and *DS *were coexpressed in different tissues. This result suggests that the two enzymes may be located in the same biosynthetic pathway. Contig00248 was classified into the CYP716 family, which is close to the CYP88 family in the phylogenetic tree of *Arabidopsis thaliana *CYP450s. Recently, CYP88D6 from *Glycyrrhiza *was identified as β-amyrin 11-oxidase, one of two characterized triterpenes hydroxylases [16]. Therefore, contig00248 is a promising candidate that may catalyze the oxidation of dammarane or protopanaxadiol.

#### Glycosyltransferases

**Figure 5:**
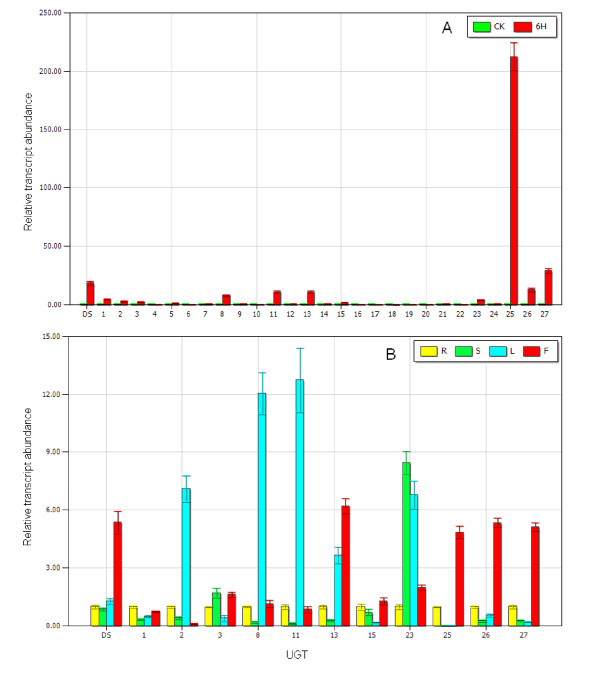
**Real-time PCR analysis of *UGT*s in MeJA-treated materials and different plant tissues**. R represents root; S represents stem; L represents leaf; F represents flower and DS represents dammarenediol-II synthase. The corresponding contigs represented by numbers 1 - 27 are listed in Additional File 6. A) Changes in gene expression of *UGT*s induced by MeJA. B) The gene expression of *UGT*s in different tissues.

Glycosyltransferases are another large multigene family in plants. In general, glycosylation is the last step in the biosynthesis of secondary metabolites. From a chemical point of view, sugar conjugation results in both increased stability and water solubility [36-38]. The 454 cDNA library contained 235 glycosyltransferase unique sequences composed of 148 contigs and 87 singletons (shown in Additional File 5). Thus far, almost all functionally characterized glycosyltransferases involved in the biosynthesis of secondary metabolites belong to UGTs and have a so-called plant secondary product glycosyltransferase (PSPG) motif, which is generally located in the C-terminal portion of the protein [37,39]. Unique sequences containing the PSPG motif were selected by a PSPG motif search in the glycosyltransferase pool. The remaining glycosyltransferases were screened again by annotation to avoid missing target sequences because most of them are not full-length sequences. The glycosyltransferases were picked up, which were annotated as the UGTs obviously involved in biosynthesis of secondary metabolites. In total, 27 *UGT*s were selected and contained reads that varied from 4 to 100 bases. Among them, a total of 11 *UGT*s were up-regulated by MeJA, whereas only three *UGT*s were obviously down-regulated in the MeJA inducibility experiment (shown in Figure 5-A). In the tissue-specific expression pattern assay, among 11 up-regulated *UGT*s by MeJA, the expression pattern of four *UGT*s showed strong similarity to that of *DS *(shown in Figure 5-B). These *UGT*s included contig01001, contig14976, contig15451, and contig16321. They were regarded as candidate *UGT*s encoding enzymes responsible for ginsenoside biosynthesis and will be the subject of further study.

## Discussion

EST analysis is one of the most popular tools for gene discovery. However, deep EST sequencing using the Sanger method is time-consuming, labor intensive, and expensive. With the development of NGS technologies, these limitations have been overcome, and EST analysis is becoming the premier choice for gene discovery on a genome-wide scale in non-model plants. In addition to advantages with regard to cost and speed, another major advantage of the NGS platform is elimination of the bacterial cloning step that can bias the composition of the cDNA library. To date, Roche GS FLX is the most widely used NGS platform for *de novo *EST sequencing. Using this technology, a number of EST libraries have been successfully constructed from plants, including maize [40], chestnut [41], olive [42], the model plant *A. thaliana *[43], and the medicinal herb *Artemisia annua *[44], as well as fish [45,46], insects [47,48], and worms [49,50].

In this study, we produced more than 200,000 HQ reads in a one-quarter run with the Roche 454 GS FLX Titanium platform. To avoid the negative effect of polyA on sequencing quality, a cDNA library preparation based on SMART technology was improved by the removal of polyA with *Bsg*I digestion, leading to an approximately 40% increase in sequencing output (data not shown). Although the Roche 454 company now prefers to resolve the polyA problem by cDNA synthesis primed with random hexamers, our study proved that enzyme digestion is at least an effective alternative way to eliminate polyA and is a promising method in 454 cDNA library preparation starting from total RNA.

Based on *de novo *sequencing and analysis of the American ginseng root, we found all of the known genes encoding enzymes involved in ginsenoside backbone biosynthesis, including *OSCs*: *DS*, and *AS*. Cyclization of 2,3-oxidosqualene is the branch point of sterol and ginsenoside biosynthesis, and OSCs play important roles in the control of carbon flux through different metabolic branches. EST number counting and real time PCR demonstrated that in the root of American ginseng, *DS *expression was highest, followed by *CAS*, and finally *AS*. This result suggested that the biosynthesis of dammarane-type ginsenosides is more active than the biosynthesis of phytosterols or oleanolic acid-type ginsenosides. Surprisingly, based on the KEGG pathway assignment, we found all of the genes involved in the biosynthesis of brassinosteroid, a phytosteroid hormone. We estimated that expression of the brassinosteroid biosynthetic genes is lower than that of the genes involved in the biosynthesis of dammarane-type ginsenosides. Therefore, these results strongly indicate that most of the genes involved in the synthesis of dammarane-type ginsenosides are contained within the 454 cDNA library.

As compared to ginsenoside backbone biosynthesis, we know little about the late stages of ginsenoside biosynthesis. This part of the pathway includes multiple oxidation and glycosylation steps catalyzed by enzymes from the CYP450 and glycosyltransferase superfamilies, respectively. These families of enzymes display a wide range of substrate specificities and are responsible for the diversity of many plant secondary metabolites. About 120 UGT genes and 272 CYP450 genes were identified in the model plant *A. thaliana*. Because of the biological, pharmacological, and agricultural importance of secondary metabolites, UGTs and CYP450s have attracted considerable interest for decades, but only a few have been characterized by traditional biochemistry and genetics. In the dammarane-type ginsenoside biosynthetic pathway, CYP450s catalyze the C12 hydroxylation of dammarenediol-II and the C6 hydroxylation of protopanaxadiol, while glycosylation generally occurs on C3, C6, and C20 of the aglycones. To date, only two CYP450s involved in triterpene saponin biosynthesis are functionally characterized: CYP88D6, a β-amyrin 11-oxidase from *Glycyrrhiza uralensis *[16] and CYP93E1, a β-amyrin and sophoradiol 24-hydroxylase from *Glycine max *[29]. Also, three UGTs in triterpene saponin biosynthesis have been identified: UGT73K1 and UGT71G1 from *Medicago truncatula *[51]and UGT74 M1 from *Saponaria vaccaria *[52]. However, all of the aforementioned enzymes are involved in oleanane-type ginsenoside biosynthesis. No CYP450s or UGTs in the dammarenediol-type ginsenoside biosynthetic pathway have been previously functionally characterized. Therefore, this study focused on the discovery of CYP450s and UGTs involved in the biosynthesis of dammarenediol-type ginsenosides, which are the major ginsenoside type in the American ginseng root.

Compared to the traditional Sanger method, 454 pyrosequencing provides a tremendous genetic resource in a fast and economical way. In total, 150 CYP450 unique sequences and 235 UGT unique sequences were found in the American ginseng cDNA library. We only found 11 *CYP450*s (one contig and 10 singletons) and two *UGT*s (two singletons) from more than 6,000 Sanger ESTs in our previous study [22]. With regard to the EST analysis of another *Panax *genus plant, *P. ginseng*, four *CYP450*s and four *UGT*s were found from about 3,000 ESTs generated from the *P. ginseng *leaf [53]; nine *CYP450*s and 12 *UGT*s were found from more than 11,000 ESTs from five ginseng library [54]. However, the large number of candidate genes produced by 454 pyrosequencing leads to difficulty in the characterization of the enzymes that are actually involved in this pathway. Therefore, MeJA inducibility experiments and tissue-specific expression pattern assays were carried out to eliminate the CYP450s and UGTs that were not involved in ginsenoside biosynthesis. After this screening, only one CYP450 and four UGT unique sequences were selected. They will ultimately be identified by their heterologous expression in *Escherichia coli *or yeast and then by an *in vitro *enzymatic assay. A similar procedure was previously described in the characterization of other CYP450s or UGTs involved in the biosynthesis of triterpene saponins[16,51]. Based on the knowledge that many secondary metabolites can be induced by MeJA and that most of the enzymes in the same pathway are coexpressed, the MeJA inducibility experiments and the tissue-specific expression pattern assays are useful in the identification of enzymes involved in the biosynthesis of secondary metabolites.

## Conclusions

American ginseng is a suitable subject for the study of triterpene saponin (ginsenoside) biosynthesis. The identification of enzymes involved in ginsenoside biosynthesis not only facilitates functional studies in the plant but also sets the stage for improving production levels in plant or microbial hosts by metabolic engineering. Based on *de novo *sequencing and analysis of the transcriptome using the Roche GS FLX Titanium platform, we found all of the known genes encoding enzymes involved in the biosynthesis of the ginsenoside backbone and established a gene pool containing 150 cytochrome P450 and 235 glycosyltransferase unique sequences. These enzymes represent two of the most important superfamilies in high plants, and a large number of them are involved in the biosynthesis of secondary metabolites. More importantly, a few candidate genes encoding enzymes responsible for hydroxylation and glycosylation in the ginsenoside biosynthetic pathway were obtained by screening with functional annotation and conducting MeJA inducibility experiments and tissue-specific expression pattern analyses. Additionally, this study represents the first example of a large-scale EST analysis from the Araliaceae family. These EST data will provide the foundation for other functional genomic research in American ginseng or its closely related species, such as *P. ginseng *and *P. notoginseng*.

## Methods

### Sample collection and preparation

Routinely, the American ginseng root (including the rhizome) is harvested between the fourth to seventh years after the initial planting for medicinal use. Therefore, four-year-old American ginseng (*P. quinquefolius *L.) was collected from the field in Huai-rou County, Beijing, China. To analyze expression induction, 200 μM MeJA (dissolved in 0.25% ethanol) was sprayed onto the leaves for 6 hours, whereas 0.25% ethanol was sprayed on the control leaves. The plant tissues were then cut into small pieces and were immediately stored in liquid nitrogen until further processing.

### RNA extraction

Total RNA was isolated using a Plant RNA Isolation Mini Kit (BioTeke, Beijing, China). RNA samples were treated with recombinant DNase I (TURBO DNase; Ambion, TX, USA) at a concentration of 1.5 units/μg of total RNA. Total RNA purity and degradation were checked on 1% agarose gels before proceeding.

### cDNA library construction and 454 sequencing

About 2 μg of total RNA extracted from the root of American ginseng was converted into cDNA using a modified SMART cDNA synthesis protocol (Clontech, CA, USA). The long poly(A/T) tails in cDNA may lead to low-quality sequencing reads from the GS FLX system. To overcome this limitation, we designed a modified poly(T) primer with a *Bsg*I site between the adaptor and the poly(T) (5'-AAGCAGTGGTATCAACGCAGAGTACT(20)VN-3'). For cDNA synthesis, this poly(T) primer was used in combination with the Clontech SMART IV primer. Double-stranded (ds) cDNA was purified with a PureLink™ PCR Purification Kit (Invitrogen, CA, USA), using Buffer HC to remove cDNAs of less than 300 bp. The cDNA was then treated overnight with *Bsg*I (NEB, MA, USA) at a concentration of 3 units/μg of cDNA. This restriction enzyme cuts within the poly(A) tail, reducing its length to 4 bases, therefore greatly increasing the quantity and quality of the 454 reads. Digested cDNA was recovered with a QIAquick PCR Purification Kit (Qiagen, Germany). About 7 μg of ds cDNA was sent to the Roche 454 Company (Branford, CT, USA) for pyrosequencing using the GS FLX Titanium Kit.

### 454 EST assembly

Using the GS FLX pyrosequencing software, we selected high-quality sequences (> 99.5% accuracy on single base reads) for further processing and assembly. Adapter trimming and poly(A/T) and short sequence (< 50 bp) removal were performed by in-house Perl scripts to obtain clean ESTs. The Newbler software (provided with the Roche GS FLX sequencer) was used for sequence assembly, and the quality score threshold was set at 40.

### Functional annotation with the BLAST program

The assembled unique transcripts were compared with the sequences in GenBank's non-redundant database using the BLASTN algorithm to find and remove ribosomal RNA sequences [55]. The remaining sequences that putatively encoded proteins were searched against the Arabidopsis protein database in the Arabidopsis Information Resource (TAIR; http://www.arabidopsis.org), the Swiss-prot protein database http://www.expasy.ch/sprot, and the NCBI non-redundant protein (Nr) database http://www.ncbi.nlm.nih.gov using the BLASTX algorithm. A typical cutoff value of E < 1.0^-5 ^was used.

### Pathway assignment with KEGG

Pathway assignments were carried out according to KEGG mapping [56]. Enzyme commission (EC) numbers were assigned to unique sequences that had BLASTX scores with cutoff values of E < 1.0e^5^, as determined upon searching the protein databases. The sequences were mapped to the KEGG biochemical pathways according to the EC distribution in the pathway database.

### PSPG motif searching

The PSPG motif exists in all known UGTs that are involved in the biosynthesis of secondary metabolites [37]. A consensus PSPG motif sequence was used to search against the glycosyltransferase database generated from the collection of 235 glycosyltransferases by the BLASTX program.

### Real-time PCR analysis

Approximately 1 μg of DNase I-treated total RNA was converted into single-stranded cDNA using a PrimeScriptTM 1st Strand cDNA Synthesis Kit (TaKaRa, Dalian, China). The cDNA products were then diluted 50-fold with deionized water before use as a template in real-time PCR. The quantitative reaction was performed on an IQ5 Multicolor Real-Time PCR Detection System (Bio-Rad, USA) using the Power SYBR Green PCR Master Mix (Applied Biosystems, CA, USA). The reaction mixture (20 μL) contained 2× Power SYBR Green PCR Master mix, 0.9 μM each of the forward and reverse primers, and 1 μL of template cDNA. PCR amplification was performed under the following conditions: 50°C for 2 min and 95°C for 30 s, followed by 40 cycles of 95°C for 15 s and 62°C for 1 min. The gene expressions of *OSC*s, *CYP450*s and *UGT*s were normalized against an internal reference gene, *glyceraldehyde-3-phosphate dehydrogenase *(*GAPDH*). For *OSC *expression analysis, *AS2 *expression in the flower tissue was arbitrarily chosen to be the calibrator of tissue gene expression. For the MeJA inducibility experiment, the expression of each gene in the uninduced material was used as the calibrator; for tissue-specific expression assay, expression in root was used as the calibrator for each gene. The relative gene expression was calculated using the 2^-ΔΔCt ^method [57]. All primers used in this study are listed in Additional File 6.

## Authors' contributions

CS conceived the study, designed and built the cDNA library, participated in data analysis, and drafted the manuscript. YL participated in sample collection, library construction, and data analysis. QW and HML participated in RNA extraction and library construction. YZS performed the real-time PCR and the corresponding data analysis. JYS and EMKL helped to conceive the study. SLC initiated the project, helped to conceive the study, and participated in the design and coordination. All authors read and approved the final manuscript.

## Supplementary Material

Additional file 1 Summary of the annotation sources for American ginseng. Click here for file

Additional file 2 Most abundant transcripts in the root of American ginseng. Click here for file

Additional file 3 **Pathway assignment based on KEGG**. A) Classification based on metabolism categories; B) Classification based on secondary metabolism categories. Click here for file

Additional file 4 **Enzymes involved in the biosynthesis of steroids based on KEGG**. Enzymes that were found in this study are marked with red rectangles. Click here for file

Additional file 5 Putative CYP450 and UGT genes in the 454 cDNA library. Click here for file

Additional file 6 Primers used in this study. Click here for file
