# Supplementary material for: De novo sequencing and analysis of the American ginseng root transcriptome using a GS FLX Titanium platform to discover putative genes involved in ginsenoside biosynthesis
Source: BMC Genomics. 2010 Apr 24;11:262. doi: 10.1186/1471-2164-11-262 (PMC2873478; doi:10.1186/1471-2164-11-262)
Supplement: Additional file 1 — Summary of the annotation sources for American ginseng. [file 1471-2164-11-262-S1.DOC]

**Additional File 1 -** Summary of the annotation sources for the American ginseng sequences

| Database | Unique sequence  (E < 1e-5) | Annotation percentage (E < 1e-5) | Unique sequence  (E < 1e-10) | Annotation percentage  (E < 1e-10) |
| --- | --- | --- | --- | --- |
| TAIR protein | 18,963 | 61.0% | 16952 | 54.5% |
| Swiss-Prot | 10,694 | 34.4% | 10694 | 34.4% |
| Nr | 20,242 | 65.1% | 18478 | 59.4% |
| Nt | 18,055 | 58.1% | 15489 | 49.8% |
| Total | 21,684 | 69.8% | 19779 | 63.6% |
