# Supplementary material for: De novo sequencing and analysis of the American ginseng root transcriptome using a GS FLX Titanium platform to discover putative genes involved in ginsenoside biosynthesis
Source: BMC Genomics. 2010 Apr 24;11:262. doi: 10.1186/1471-2164-11-262 (PMC2873478; doi:10.1186/1471-2164-11-262)
Supplement: Additional file 2 — Most abundant transcripts in the root of American ginseng. [file 1471-2164-11-262-S2.DOC]

**Additional File 2 - Most abundant** transcripts in the root of American ginseng

| **contig ID** | **No. of ESTs**  **In the contig** | **putative function** | **Accession No.** | **source** | **E-valule** |
| --- | --- | --- | --- | --- | --- |
| contig01059 | 3102 | Regulator of ribonuclease-like protein 1 | sp|Q9M8R9|RRAA1_ARATH | *Arabidopsis thaliana* | 3.00E-42 |
| contig15901 | 2608 | Ribonuclease-like storage protein | sp|P83618|RN28_PANGI | *Panax ginseng* | 9.00E-22 |
| contig15257 | 2605 | Panax quinquefolius specific abundant protein-like protein 1 mRNA, complete cds | gb|EU274652.1| | *Panax quinquefolius* | 2.00E-88 |
| contig16371 | 2111 | Panax ginseng major latex-like protein (mlp151) mRNA, complete cds | gb|EU939308.1| | *Panax ginseng* | 2.00E-20 |
| contig16583 | 1963 | Panax quinquefolius hypothetical protein LBL3 mRNA, complete cds | gb|EU136392.1| | *Panax quinquefolius* | 2.00E-85 |
| contig00296 | 1741 | no hit |  |  | -- |
| contig00210 | 1677 | Sucrose synthase isoform 1 | sp|P49035|SUS1_DAUCA | *Daucus carota* | 0 |
| contig15623 | 1578 | Catalase | sp|O24339|CATA_SOLAP | *Soldanella alpina* | 0 |
| contig16037 | 1564 | Ribonuclease 1 | sp|P80889|RNS1_PANGI | *Panax ginseng* | 3.00E-42 |
| contig14300 | 1556 | 1,4-alpha-glucan-branching enzyme | sp|P30924|GLGB_SOLTU | *Solanum tuberosum* | 0 |
| contig16257 | 1414 | Ribonuclease-like storage protein | sp|P83618|RN28_PANGI | *Panax ginseng* | 1.00E-13 |
| contig15946 | 1398 | no hit |  |  | -- |
| contig00290 | 1266 | Peroxidase 42 | sp|Q9SB81|PER42_ARATH | *Arabidopsis thaliana* | 1.00E-147 |
| contig15733 | 1118 | Omega-6 fatty acid desaturase, endoplasmic reticulum isozyme 2 | sp|P48631|FD6E2_SOYBN | *Glycine max* | 1.00E-115 |
| contig16459 | 1112 | Panax ginseng major latex-like protein (mlp151) mRNA, complete cds | gb|EU939308.1| | *Panax ginseng* | 4.00E-20 |
| contig00091 | 1080 | Uncharacterized mitochondrial protein AtMg00030 | sp|P93276|M030_ARATH | *Arabidopsis thaliana* | 2.00E-27 |
