# Supplementary material for: De novo sequencing and analysis of the American ginseng root transcriptome using a GS FLX Titanium platform to discover putative genes involved in ginsenoside biosynthesis
Source: BMC Genomics. 2010 Apr 24;11:262. doi: 10.1186/1471-2164-11-262 (PMC2873478; doi:10.1186/1471-2164-11-262)
Supplement: Additional file 3 — Pathway assignment based on KEGG. A) Classification based on metabolism categories; B) Classification based on secondary metabolism categories. [file 1471-2164-11-262-S3.DOC]

**Additional File - 3** **Pathway assignment based on KEGG.** A) Classification based on metabolism categories; B) Classification based on secondary metabolism categories
