# Supplementary material for: De novo sequencing and analysis of the American ginseng root transcriptome using a GS FLX Titanium platform to discover putative genes involved in ginsenoside biosynthesis
Source: BMC Genomics. 2010 Apr 24;11:262. doi: 10.1186/1471-2164-11-262 (PMC2873478; doi:10.1186/1471-2164-11-262)
Supplement: Additional file 4 — Enzymes involved in the biosynthesis of steroids based on KEGG. Enzymes that were found in this study are marked with red rectangles. [file 1471-2164-11-262-S4.DOC]

**
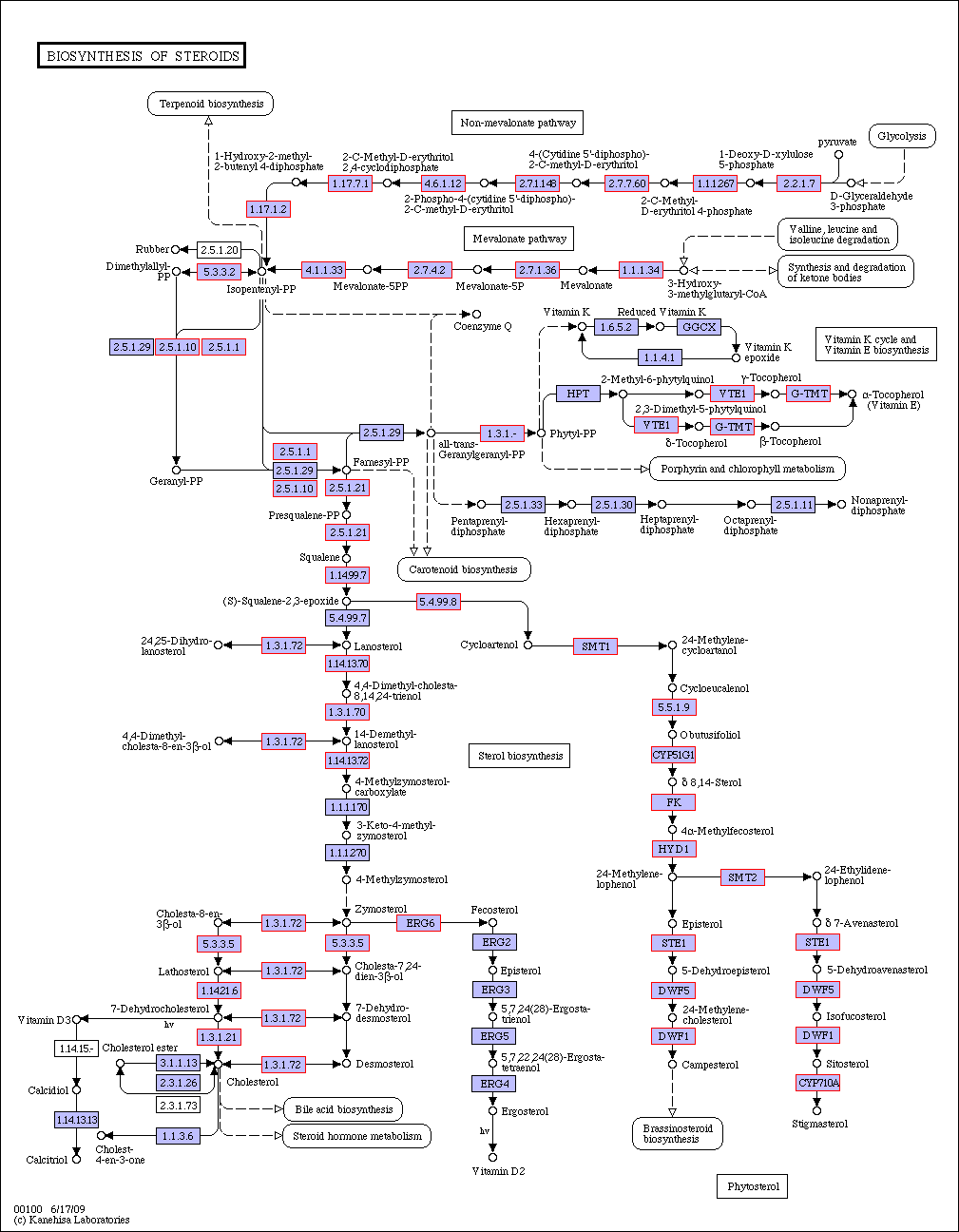
**

### Additional File 4 - Enzymes involved in the biosynthesis of steroids based on KEGG. The enzymes that were found in this study were marked by red rectangles.
